# Internal adaptation and micromorphological analysis of a new self-cure resin composite

**DOI:** 10.4317/jced.62900

**Published:** 2025-08-01

**Authors:** Eman H Albelasy, Ahmed Gamal Raghip, Hoda Saleh Ismail

**Affiliations:** 1 Faculty of Dentistry, Conservative Dentistry Department, Mansoura University, Egypt; 2 Faculty of Oral and Dental Medicine, Restorative Dentistry Department, Alsalam University, Egypt

## Abstract

**Background:**

Enhancing the adaptation and durability of composite restorations remains a major challenge in modern adhesive dentistry. Recently, a new self-cured resin composite has gained attention with the potential to improve interfacial adaptation and reduce polymerization shrinkage stress, offering an alternative to conventional light-cured systems.

**Material and Methods:**

Sixteen freshly extracted human molars were prepared with standardized Class II cavities and randomly assigned to two groups (*n*=8). The first group was restored using a self-cure bulk-fill resin composite (Stela Automix, SDI Ltd., Australia) with its respective primer, while the second group received an injecTable resin composite (G-aenial Universal InjecTable, GC Corporation, Japan) with a universal adhesive. Restorative procedures followed manufacturers’ instructions. The restorations were finished and polished, before being stored in distilled water at 37°C for six months to simulate aging conditions. After storage, specimens were sectioned longitudinally and analyzed using environmental scanning electron microscopy to evaluate internal adaptation, and interfacial gap measurements were recorded.

**Results:**

An independent Sample T-test showed a statistically significant difference in interfacial gap (IG%) between the two restorative systems, with the self-cure composite showing more favourable outcomes. For stela, the IG% was13.5±6.1 while for InjecTable composite IG%=28.4±13.3.

**Conclusions:**

The Stela primer and composite used in this study demonstrated superior internal adaptation compared to the light-cured control, suggesting they could be a viable alternative, particularly for deep gingival margins or situations where light curing is inaccessible.

** Key words:**Self-cure composite, internal adaptation, SEM, curing mode, Universal adhesive.

## Introduction

Resin composites are commonly used as restorative materials due to their excellent aesthetics and their ability to bond with tooth structure through an adhesive [1]. However, when resin composite monomers are converted into a polymer network, it results in bulk contraction, causing a volumetric polymerization shrinkage of 1.67%-5.68%. The shrinkage that occurs during “rapid photo-polymerisation,” along with the high elastic modulus of the composite, creates internal stresses that can weaken the bond between the composite and dental tissues (such as dentine and enamel). This results in gap formation and a reduction in bonding effectiveness [2-4].

Light-cured resin-based composites (RBCs) typically rely on camphorquinone as the photo-initiator, paired with a tertiary amine as the co-initiator. When the RBC is exposed to adequate light at the appropriate wavelengths, the photo-initiator and tertiary amine react to generate free radicals, which then break the double bonds and start the polymerization process [5]. Tertiary amines enhance the depth of cure and accelerate the polymerization process in light-cured resin-based composites (RBCs) [6], but the combination of the photo-initiator and tertiary amine tends to have poor color stability [7]. Inadequate light exposure during curing of RBC can lead to a shallow depth of cure [8], reduced mechanical properties [9], lower bond strength, and an increased risk of fracture or failure of the restoration [10-12].

Self-cured or dual-cured resin-based composites (RBCs) offer an essentially unlimited depth of cure and may be more suiTable for use in deep cavities compared to light-cured RBCs [13,14]. Self-cured RBCs do not require light exposure since they lack photoinitiators in their formulation, making them ideal for situations where a curing light is unavailable. Additionally, they have a slower polymerization rate [13] and, when combined with newer monomers and primers [13,14], may achieve a higher degree of conversion. As a result, using a self-cured RBC can potentially improve the overall quality of the final restoration [15].

A new chemically cured (self-cured), bulk-fill restorative material has recently been launched on the market (Stela, SDI, Victoria, Australia). Available in two application forms—Stela Automix and Stela Capsule—this product has an adhesive property that eliminates the need for light-curing, as it polymerizes when it comes into contact with the restorative material. Several *in vitro* studies have assessed this new material, showing promising results [16-18]. This material includes various fillers like strontium fluoro-alumino-silicate glass, ytterbium trifluoride agglomerates, silica, and calcium aluminate. While some might question whether this restorative material qualifies as a compomer, the manufacturer classifies STELA as a new-generation, resin-based bulk-fill restorative material with distinct chemico-physical properties [17].

Internal adaptation describes how well a restoration adapts internally to the tooth substrate [19]. Aging factors like water storage, mechanical stresses, and temperature fluctuations negatively impact the tooth–restoration interface, causing the development of internal gaps [20-22]. The degradation of the tooth–restoration interface occurs primarily through chemical or mechanical processes. Initially, it is exposed to water and enzymes, and later, enzymes released from the dentin matrix may lead to hydrolysis of resin or collagen, along with the breakdown of adhesives and composite resin components [23,24]. Additionally, water can induce “plasticization” of the resin, which involves swelling and a reduction in the friction between polymer chains, resulting in diminished mechanical properties of the polymer matrix [25].

Despite the extensive research on the properties and behaviour of light-cured resin-based composites, there is a limited focus on the characteristics of self-cured resin composites, particularly in terms of their interface with dentine. While the shrinkage and stress associated with polymerization in light-cured materials are well-documented, similar evaluations for self-cured materials still lacking. Therefore, this study aims to address this gap by investigating the internal adaptation and micromorphological features of self-cured resin composites, specifically focusing on the interface between the material and dentine.

## Material and Methods

1. Sample size 

The sample size was calculated using G.Power software (Version 3.1.9.7; GPower, Kiel, Germany). This calculation was based on a prior study that compared the marginal adaptation of self-cure composite, bulk-fill composite, and conventional composite [17]. A rough estimate of Cohen’s D was made, assuming a moderate effect size to determine the minimal detecTable effect size. The parameters used included an effect size of 0.68, 80% power, a significance level of 0.05, and equal sample distribution across groups. The calculated sample size was 8 samples per group.

2. Materials 

This study employed two restorative materials with comparable viscosities: a self-cure bulk-fill resin composite (Stela Automix, SDI Ltd., Australia) and an injectable resin composite (G-ænial Universal Injectable, GC Corporation, Tokyo, Japan). The bulk-fill composite was used in conjunction with Stela primer, while a universal adhesive (G-PERMIO Bond, GC Corporation, Tokyo, Japan) was applied with the injectable composite. Detailed specifications for the materials are provided in  Table 1. All materials in this study were utilized in accordance with the manufacturer’s instructions.

2.1 Methods

For this study, sixteen freshly extracted human molars, which were periodontally compromised but otherwise healthy, were chosen. The teeth were collected with written consent from patients who were informed that the teeth would be used for research purposes, following approval from the University’s ethical committee (SUE 011902252). After extraction, the teeth were stored in a 0.1% thymol solution at 4ºC for no longer than one month before being used.

Before use, the teeth were thoroughly cleaned to remove any calculus using an ultrasonic scaler. The roots were then embedded in self-curing acrylic resin, extending up to 3.0 mm below the cementoenamel junction (CEJ). A single trained operator with eight years of clinical experience prepared the cavities using a flat-ended cylindrical diamond bur with a high-speed handpiece and continuous water irrigation. The cavity preparation was completed using a yellow-coded diamond bur, with burs being replaced after every five preparations.

Standardized, box-shaped Class II cavities were prepared on the proximal surfaces of the teeth, with the gingival margin positioned 1.0 mm below the cementoenamel junction. The cavity dimensions were as follows: occlusally, the buccolingual dimension was 3.00 mm, with a depth of 3.00 mm. For the proximal box, the axial-pulpal depth was 1.5 mm, measured using a graduated periodontal probe, and the buccolingual dimension of the box was approximately 2.5 mm. The cavity walls were not beveled. Cavity dimensions were measured using a graduated dental probe, and any cavities exceeding these dimensions by more than ±15% were excluded. The teeth were randomly assigned into two groups (*n*=8) based on the type of restorative system using a computer-generated random number sequence in Microsoft 365 Excel.

2.2 Restorative steps 

For the self-cure restorative system (Stela), enamel margins were selectively etched with 37% phosphoric acid (N-Etch, Ivoclar Vivadent, Amherst, NY, USA) for 15 seconds, rinsed with water for the same duration, and gently dried with oil-free air without causing desiccation. A Tofflemire matrix band (Universal #1, JR Rand, NY, USA) was used to adapt the restorations to the cavity margins. Two drops of primer were dispensed into a plastic mixing well and vigorously rubbed against the cavity preparation for 10 seconds using a disposable applicator brush. Afterward, it was gently air dried for 2-3 seconds. The primer was left to sit for 5 seconds. Stela Automix was applied to the cavity, which was overfilled and meticulously sculpted. The restoration was allowed to set for 4 minutes. For the injectable composite, a universal adhesive (G-PERMIO Bond, GC Corporation, Tokyo, Japan) was applied after selective enamel etching for 15 seconds. The adhesive was rubbed onto the cavity surface, followed by a 10-second wait, air-thinned for 5 seconds, and then light cured using an LED curing light (Elipar Deep Cure, 3M ESPE, St. Paul, MN, USA) operating at 1,000 mW/cm². The injectable composite was applied in two increments and light cured for 20 seconds with the Elipar Deep Cure (3M ESPE, St. Paul, MN, USA) operating at 1,000 mW/cm². Additional curing was performed after removing the matrix band. The light-curing device was checked with a radiometer (Bluephase Meter II, Ivoclar Vivadent). All specimens were stored in distilled water at 37°C for 24 hours in an incubator prior to the finishing and polishing procedures. The restorations were finished with aluminum oxide discs (Sof-Lex discs, 3M ESPE, MN, USA) and polished using a 3-step polishing system (Kenda, Coltene/Whaledent, Altstätten, Switzerland).

2.3 Water storgae 

All samples were stored in distilled water for 6 months in an incubator at 37ºc to challenge the adhseive interface.

2.4 Scanning Electron Microscopy-Internal adaptation 

After 6 months of storage in distilled water, the restored teeth were sectioned longitudinally in mesiodistal direction under copious water cooling using a water-cooled diamond blade (Isomet Diamond Wafering Blade, no. 11–4244, Buehler Ltd., Lake Buff, IL, USA). Each tooth was sectioned into two halves. One was used for internal adaptation, and the other half for micromorphological analysis. The halves were polished using a sequence of aluminium-oxide abrasive discs from coarse (50–90 µm) to superfine (1–7 µm) (Sof-Lex Polishing discs (3M, St. Paul, MN, USA). The finishing and polishing process was conducted with copious water irrigation. Specimens were then cleaned in an ultrasonic bath for 10 minutes to remove any debris. Analysis of any internal interfacial gaps was conducted using an environmental scanning electron microscopy (Quanta 250 FEG, Netherlands).

Micrographs were taken at standardized magnification (180x, 500x), in order to document the bonded internal interface. The values of the 4 internal surfaces per specimen were averaged. The dentin-restoration interface was marked out to observe and measure the length of the interfacial gaps. ImageJ software (National Institutes of Health, Bethesda, MD, USA) was used for calibration and to measure the length of debonded segments along the designated dentin-restoration interface. The scale bar from the SEM image was utilized for calibration, and the lengths of the debonded segments were recorded in micrometers. The total length of the unbonded interface was calculated by summing the individual gaps, and the interfacial gap percentage (IG%) was determined using the formula: ((unbonded length/total length) x 100 = IG%) [26]. To ensure accuracy, a single experienced operator performed all measurements. The data were analyzed using SPSS software (version 20, IBM, Chicago, IL, USA). An independent samples t-test was conducted to evaluate the impact of the restorative system on interfacial gap percentage, following a Shapiro-Wilk test for normality. A 95% confidence level was maintained, with the significance level set at 0.05.

4. Micromorphological analysis 

For the micromorphological analysis, each slab was polished wet with 600-, 1200-, and 2000-grit SiC paper. Afterward, the specimens were further polished with soft cloths and diamond pastes of progressively finer abrasiveness. The slabs were then etched with a 10% orthophosphoric acid solution for 10 seconds to remove mineral content, rinsed with water, and soaked in 5% sodium hypochlorite for 5 minutes to eliminate exposed collagen from the dentin surface. Finally, the specimens underwent an ultrasonic cleaning in distilled water for 10 minutes and were left to dry overnight at 37°C.

## Results

1. Internal Adaptation 

**Figure 1:**
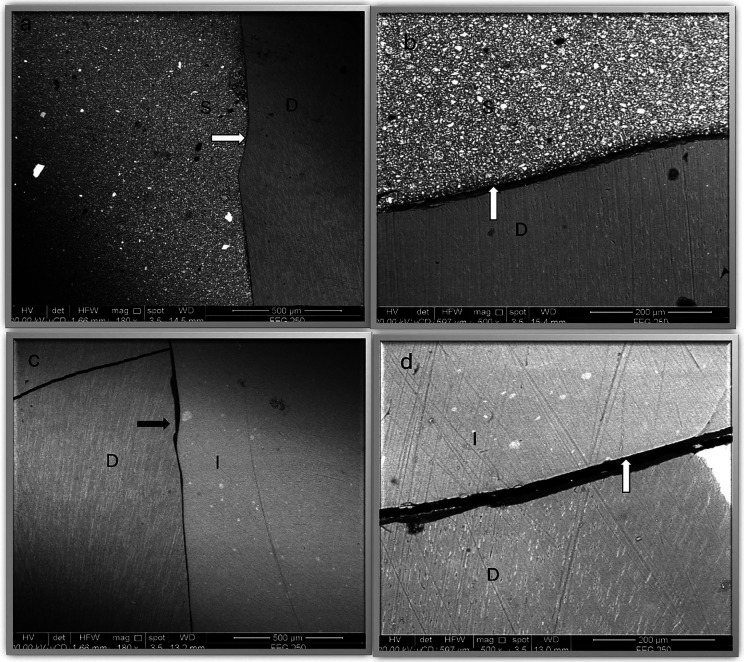
Representative SEM images of internal adaptation evaluation of the tested materials. a, and c: Stela, and G-ænial Universal Injectable at ×180 showing good internal adaptation, with IG%=0. b, and d: showing Stela and injectable at X 500 with interfacial gap (The arrow is pointing at the gap).

An independent Sample T-test showed a statistically significant difference in IG% between the two restorative systems, with the self-cure Stela showing more favourable outcomes. For stela the IG% was13.5±6.1 while for Injectable compoiste IG%=28.4±13.3 The results are presnetd in  Table 2. Representative cross-sectional images for both restorative materials with IG% = 0 are shown in Fig. 1a,c), while interfacial gaps are illustrated in Fig. 1 (b,d).

2. Micromorphological analysis 

**Figure 2:**
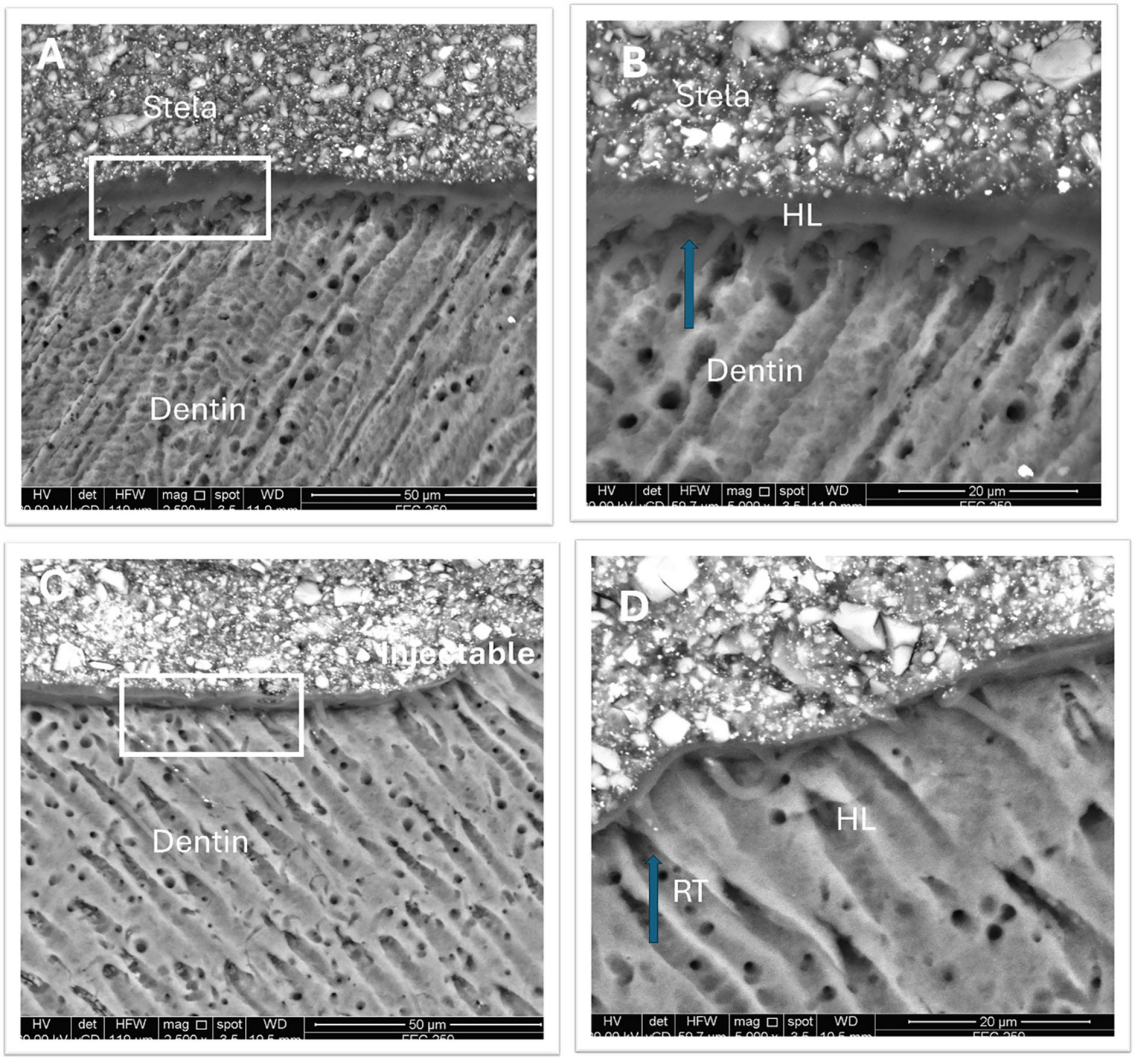
Representative SEM micrographs of the resin-dentin interface formed by the Stela primer + composite after six months of storage in distilled water are shown at (A) 2500× magnification and (B) 5000× magnification (HL: hybrid layer, RT: resin tags). Similarly, SEM images of the resin-dentin interface created using the G-Premio Bond multi-mode adhesive in selective enamel-etch mode, combined with an injectable nano-hybrid composite, after six months of distilled water storage are shown in (C) and (D).

Representative regions of the resin-dentin interfaces are displayed in Figure 2. Both restorative materials produced thin hybrid layers with visible resin tags. The resin tags were longer when using the Injectable nanohybrid resin composite material with the Universal adhesive (C&D), whereas for Stela (A &B), they appeared shorter and thicker.

## Discussion

Enhancing the adaptation and durability of composite restorations remain one of the key challenges in modern adhesive dentistry and dental research. Previous research has shown that the quality of the tooth-restoration interface is influenced by various procedural and material factors, including the composition and type of adhesive system, etching method, the type and shrinkage stress of the restorative material, cavity size and type, as well as the insertion and polymerization techniques [27-29]. This study compared the internal adaptation of a new self-cure resin composite with its associated primer to a highly filled flowable composite combined with a multi-mode adhesive used in selective-enamel etch mode. The findings reject the null hypothesis, demonstrating a significant difference between the two, with the self-cure composite showing better internal adaptation.

There is evidence suggesting that dual- and self-cured materials produce less stress compared to light-cured materials [30,31]. When the resin-based composite (RBC) has a slower polymerization rate, it typically generates lower stress due to a longer pre-gel phase and slower initiation, which may result in reduced shrinkage stress [31], and a consequent reduction in gap formation. Furthermore, the synergistic bonding factor likely played a role in reducing excessive stress at the interface, which was further supported by the use of the Stela Primer, as it self-polymerized alongside the Stela restorative, as has been previously reported [17]. Furthermore, in a previous study [5], the Stela Primer was found to initiate the polymerization reaction from the cavity walls and bottom. The finding that polymerization begins at the tooth/primer/RBC interface rather than in the bulk of the RBC could be responsible for minimizing gap formation at the tooth/RBC interface.

The results of this study indicate the ability of the stela primer and composite to form a hybrid layer with uniform thickness, albeit with short resin tags and partially occluded dentinal tubules. Stela primer is HEMA-free but contains the monomer GDMA, (molecular weight: 228.24 g/mol) which is hydrophilic nature, similar to HEMA (molecular weight: 113.14 g/mol). However, GDMA differs by containing two methacrylate functional groups in addition to a hydroxyl unit. Which has been previously shown to decrease water sorption and solubility [32]. This is likely due to its dimethacrylate structure, which promotes additional polymer crosslinking, forming a stable polymer network that minimizes the risk of hydrolysis [32]. It is worth mentioning that the dentine was not previously treated, which explains the presence of partially occluded dentinal tubules. Stela rimer contains the monomer 10-MDP which is able to chemically interact with the remaining minerals in dentin.

G-ænial Universal Injectable is a relatively new material that, despite the terminology used, is still essentially a highly filled flowable composite. According to a recent study [33], it has a greater depth of cure than conventional flowable composites but a lower depth of cure than bulk-fill composites. Therefore, it was applied using a layering technique. The enhanced handling of flowable resin composite is undoubtedly a key clinical advantage, particularly when working with dentin cervical margins [34]. However, a previous study [35], showed that conventional composite had similar adaptation to flowable showing that that this factor was not as critical in this controlled in-vitro setting. on the other hand, the amount of filler and the different monomers contained in tested materials might have led to the difference. The percentage of the interfacial gap in this composite can be attributed to its high shrinkage rate, which is likely due to its lower filler content, with a filler volume of 50% and a filler weight of 69% [36].

The resin-dentine interface of specimens created using injectable composite with multi-mode adhesive (G-Premio bond) exhibited a thinner hybrid layer, and a high percentage of interfacial gaps. These defects were most likely caused by excessive composite shrinkage, which the adhesive in dentine self-etch mode was unable to adequately compensate. Furthermore, evident signs of aging were frequently observed at the resin-dentine interface, as well as within the adhesive and hybrid layer, after 6 months of storage in water. This outcome is likely due to significant water sorption by the one-bottle “simplified” adhesive which triggered extensive hydrolytic degradation at the resin-dentine bonding interface [37,38]. The bonding performance of a multi-mode adhesives relies on the ability of its functional and/or acidic monomers to interact with the smear layer and the underlying mineralized dentin [38]. The Universal adhesive used in this study contains 4–methacryloxyethyl trimellitic acid (4-MET) as a functional monomer. It has been reported [39], that 4-MET has a strong chemical bonding potential to calcium-containing substrates, similar to10- MDP.

Furthermore, for Class II cavities with proximal gingival margins extended to the dentine, it is common practice to perform additional photo-polymerization from both the buccal and lingual sides after removing the matrix band. This compensates for the increased distance between the light-curing device tip and the composite layer at the gingival margin [40]. However, using a light-cured adhesive in deep subgingival areas poses a risk of incomplete polymerization, which could affect the long-term stability of the adhesive restorations in such cases [41]. It is also worth mentioning that the universal adhesive used in this study is HEMA free but contains acetone which has the potential to enhance water sorption and subsequent hydrolysis [42,43].

The FDI has highlighted the importance of continued research to improve the overall properties of dental materials, aiming to enhance their clinical performance and cost-effectiveness [44]. Therefore, further studies are needed to evaluate the wear resistance, polymerization shrinkage, shrinkage stress, and bond strength of stela restorative/primer. Additionally, this study has certain limitations. Although six months of water storage subjects the adhesive interface to degradation, it may not fully replicate the long-term aging processes in the oral environment. Moreover, the absence of mechanical cycling, which can further challenge the adhesive interface, is another limitation.

## Conclusions

The Stela primer and composite used in this study demonstrated superior internal adaptation compared to the light-cured control, suggesting they could be a viable alternative, particularly for deep gingival margins or situations where light curing is not accessible.

## Figures and Tables

**Table 1:** Materials used in the study.

Material	Manufacturer	Composition	Lot Number	Specification
Stela Primer	SDI, Victoria, Australia)	Methyl ethyl ketone (10-30 %), 4-methacryloxyethyl trimellitic anhydride (10-30 %), acrylic monomer (10-30 %), 10-methacryloyloxydecyl dihydrogen phosphate (10- MDP; 10-30 %) and diurethane dimethacrylate (DUDMA; 10-30 %) (**)	1213099	A self-cure primer
Stela automix	SDI Ltd., Australia	Organic matrix (***): DUDMA (10-25 %), glycerol dimethacrylate (GDMA; 5-10 %), ytterbium fluoride (3-7 %) and 10-MDP (1-5 %). Filler content (****): Fluoro- alumino-silicate glass: mean particle size 4.0 m (distribution range approx. 2 to 8 m) and Barium-alumino- borosilicate glass: mean particle size 2.8 m (distribution range approx. 2 to 5 m). Filler loading: 61.2 wt% (36.4 vol%)	1216386A	Self-cure bulk-fill composite
G-Ã¦nial Universal Injectable	GC Corporation, Tokyo, Japan	4,4-Isopropylidenediphenol, 2-methylprop-2-enoic acid, (octahydro-4,7-methano-1H-indenediyl) bis (methylene) bismethacrylate, 2,2-dimethyl-1,3-propanediyl bismethacrylate, 2,2-ethylenedioxydiethyl dimethacrylate, UDMA, 2,6-di-tert-butyl-p-cresol, 2-(2H-benzotriazol-2-yl)-p-cresol, 1,3,5-Triazane-2,4,6-triamine, diphenyl (2,4,6-trimethylbenzoyl) phosphine oxide, silica, barium glass (fillers 69wt%)	2212221	Nano-hybrid high-strength low-viscosity composite
G-PERMIO Bond	GC Corporation, Tokyo, Japan	10- ethacryloyloxydecyl dihydrogen phosphate, 4-methacryloxyethyl trimellitate, methacryloyloxyalkyl thiophosphate methylmethacrylate, methacrylate monomer, acetone, water, silica, initiator.	2312021	Universal adhesive

**Table 2:** Results of Independent Sample T-Test.

Group	Mean±SD	P. Value
Stela	13.5±6.1	.013
Injectable		

## Data Availability

The datasets used and/or analyzed during the current study are available from the corresponding author.
